# Cardiovascular Care of Tactical Athletes: Have We Landed Yet?

**DOI:** 10.1016/j.jacadv.2024.101236

**Published:** 2024-08-30

**Authors:** Bradley J. Petek, Eugene H. Chung, Jonathan H. Kim, Denise L. Smith, Stefanos N. Kales, Aaron L. Baggish, M. Alaric Franzos, Mark C. Haigney, Benjamin D. Levine, Elizabeth H. Dineen

**Affiliations:** a Sports Cardiology Program, Knight Cardiovascular Institute, Oregon Health & Science University, Portland, Oregon, USA; b Massachusetts General Hospital, Harvard Medical School, Boston, Massachusetts, USA; c Emory Clinical Cardiovascular Research Institute, Emory University School of Medicine, Atlanta, Georgia, USA; d Department of Health and Human Physiological Sciences, Skidmore College, Saratoga Springs, New York, USA; e Occupational Medicine, Cambridge Health Alliance/Harvard Medical School, Boston, Massachusetts, USA; f Department of Environmental Health, Harvard T.H. Chan School of Public Health, Boston, Massachusetts, USA; g Cardiovascular Performance Program, Massachusetts General Hospital and Harvard Medical School, Boston, Massachusetts, USA; h Department of Cardiology, University of Lausanne, Lausanne, Switzerland; i Department of Sports Sciences, University of Lausanne, Lausanne, Switzerland; j Military Cardiovascular Outcomes Research, Uniformed Services University, Bethesda, Maryland, USA; k Institute for Exercise and Environmental Medicine, The University of Texas Southwestern Medical Center, Dallas, Texas, USA; l Department of Cardiovascular Medicine, Mayo Clinic Florida, Jacksonville, Florida, USA

**Keywords:** firefighter, police, military, pilot, cardiovascular

Tactical athletes (TAs) are those “whose occupations have significant physical fitness requirements…potential exposure to life-threatening situations”^1^ all while serving others. Accordingly, there are numerous occupations that may be considered TAs, including military members, law enforcement, firefighters (both structure and wildland), emergency medical services personnel, and astronauts. These athletes may encounter intense mental and physical stressors (often requiring significant muscular work including speed and burst performance while wearing personal protective clothing and carrying heavy equipment), extreme environmental conditions (eg heat, cold, altitude), unpredictable work schedules and most importantly, the lives of others may be dependent on the TA. Furthermore, teams of TAs may include individuals across a wide spectrum of age and fitness. These stressors and characteristics represent unique differences between TAs and athletes participating in competitive sports. There has been a paradigm shift to adopt a shared decision-making approach for eligibility decisions among athletes with cardiovascular conditions who participate in competitive sports. However, this approach may not be feasible for TAs where the lives of others or the success of missions may depend on an individual TA’s ability to safely perform duty. While significant prior research has defined the impact of high levels of exercise on the cardiovascular system and the safety of exercise among competitive athletes with cardiovascular disease (CVD), there has been little scientific inquiry on the cardiovascular care of TAs. In the following sections, we summarize prior cardiovascular research focused on TAs and identify key areas for future scientific inquiry (Table 1).

**Table 1:** Top 10 Areas of Future Scientific Inquiry Among Tactical Athletes

1. The creation of large registries and surveillance studies to characterize the incidence and outcomes among TAs with CVD
2. The formation of guidelines and scientific statements by cardiovascular societies to help in the creation of cardiovascular protocols and screening programs specific for TAs
3. The causes of SCA/D should be characterized among TA groups (using an expert panel for adjudication to review cardiac autopsies if not performed at a centralized cardiovascular pathology center) to inform screening practices
4. Policies to include postmortem genetic testing in the routine evaluation of young TAs should be considered to improve diagnostic yield and inform the need for cascade screening
5. Future research should prioritize determining the incidence of non-SCA/D cardiovascular conditions that may lead to significant safety concerns among TAs (eg, CAD, arrhythmias, myocardial infarction, heart failure)
6. Focus should be placed on the safety of return to duty following treatment among TAs with cardiovascular symptoms or conditions
7. Nontraditional risk factors, such as environmental exposures, should be considered to assess for possible cardiotoxic risks
8. Research is needed to understand the safety and cardiovascular effects of current and future substances used to improve occupational performance among TAs
9. Well-designed implementation studies are needed to rigorously test the effects of treatment programs to reduce risk from traditional and nontraditional cardiovascular risk factors among TAs
10. The diagnostic and prognostic yield for individual tests included in PPCS are needed to inform practice (eg, resting ECG, TTE, exercise tests)

CAD = coronary artery disease; CVD = cardiovascular disease; ECG = electrocardiogram; PPCS = preparticipation cardiovascular screening; SCA/D = sudden cardiac arrest/death; TA = tactical athlete; TTE = transthoracic echocardiogram.

Unlike many other disciplines within cardiology, most research studies on TAs come from small observational cohorts from individual centers, with few prospective registries. As a result, there are significant knowledge gaps on the optimal cardiovascular care of TAs, and most recommendations from individual TA governing/advising bodies are derived from expert opinion rather than robust scientific evidence. Cardiovascular society scientific statements and guidelines on best practices for TAs do not currently exist. There is also imbalance in the published research for different TA groups, with many studies among military and firefighter populations, and few studies among other occupations such as law enforcement. Overall, large-scale prospective registries are needed to advance knowledge on the cardiovascular care of TAs and thus, enable evidence-based recommendations in TA-specific scientific statements and guidelines (Table 1, Number 1 and 2).

Sudden cardiac arrest/death (SCA/D) is the most consequential cardiovascular event among TAs as there may be a significant impact on the survival of others or the success of strategic missions. Prior studies have found that the leading medical cause of duty-related death among TAs is CVD, and TAs are more likely to sustain a SCA/D event while performing strenuous duties.^2^^,^^3^ As there is a wide age spectrum within different TA service occupations, causes of SCA/D may vary from underlying genetic and congenital conditions as are more common among young populations (age <35 years) to underlying atherosclerotic coronary artery disease (CAD), the predominant cause of SCA/D among older TAs.^2^^,^^3^ Numerous TA organizations do not have centralized cardiovascular pathology divisions to adjudicate causes of death among cases of sudden cardiac death (SCD). Therefore, future epidemiological studies should be designed with an adjudication process to ensure a diagnosis listed on an autopsy report is the actual cause of death to better understand the leading causes of SCD among TAs to better inform cardiovascular screening programs (Table 1, Number 3).

Postmortem genetic testing can increase the diagnostic yield and inform cascade screening following a SCD event, especially among young individuals with autopsy-negative sudden unexplained death. To date, there have not been large-scale studies utilizing postmortem genetic testing to better delineate the causes of SCD among TA cohorts. Postmortem genetic testing is available as a covered benefit for military decedents through the Defense Health Agency Genetics Reference Laboratory, but access to testing may not be widely available for other TA groups. Among military members, when autopsies are performed outside the military health system, the opportunity to leverage this capacity for postmortem genetic testing is often overlooked. Future work is needed to improve the access and utilization of postmortem genetic testing among TAs to inform future research and clinical care (Table 1, Number 4).

Other cardiovascular conditions causing acute symptoms or temporary incapacitation may pose significant safety concerns for the TA. These conditions may include, but are not limited to, acute arrhythmias, non-arrhythmic syncope, myocardial infarction, or acute heart failure. As an example, neurocardiogenic syncope is a relatively common and typically benign finding among young people. However, if a fighter pilot experiences neurocardiogenic syncope in the air, this may lead to a fatal outcome (as opposed to other tactical duties which may only warrant further workup for recurrent neurocardiogenic syncope). There are very limited available data on the incidence of cardiovascular conditions among TAs, and subsequent safety of return to duty after appropriate treatments (Table 1, Number 5 and 6). Data on the use of coronary artery calcium (CAC) scores as a screening test in firefighters have suggested that firefighters may have higher-than-expected amounts of CAC, which could in theory lead to worse cardiovascular outcomes.^4^ Preliminary studies assessing outcomes among United States military pilots diagnosed with atrial fibrillation have found that 40% were able to safely redeploy after medical evaluation and treatment.^5^ Not all cardiovascular conditions will lead to significant safety concerns and morbidity, but it is critical to conduct further research to better understand their prevalence, risks of SCA/D or other sudden incapacitation, best management practices, and safety upon return to duty among all conditions.

Preventive efforts, especially for CVD, are essential to avoid deleterious sequelae associated with acute cardiovascular events among TAs. Multiple prior studies have found that TA cohorts may have higher-than-expected cardiovascular risk factors, such as an increased prevalence of hypertension among firefighters.^6^ While previous research has begun to characterize the prevalence of traditional cardiovascular risk factors, it is important to also consider nontraditional exposure-related risk. Exposure to extreme environmental conditions or noxious chemical agents could, in theory, lead to an increase in cardiovascular risk. For instance, exposure to air pollution and particulate matter has been associated with increased cardiovascular risk and may affect groups such as firefighters and military recruits.^7^ Also, astronauts may be exposed to significant radiation exposure during spaceflight, which may accelerate atherosclerosis. Care should be taken when designing studies that not only control for traditional cardiovascular risk factors but also attempt to directly measure occupational exposures to prove the presence or absence of cardiotoxic effects (Table 1, Number 7). TAs occasionally take performance-enhancing supplements (eg stimulants), though supplements are not regulated, during sustained operations, while avoiding banned substances; further research is needed to document these cardiovascular effects (Table 1, Number 8). Another key area of future research is the implementation of programs to reduce cardiovascular risk. With multiple prior studies defining the prevalence of cardiovascular risk factors, implementation studies are needed to characterize the best risk reduction strategies in occupational settings (Table 1, Number 9).

Preparticipation cardiovascular screening (PPCS) to test for diseases associated with adverse cardiovascular outcomes may decrease risk among TAs. Among the different TA organizations, there is a wide array of PPCS methods that include the history and physical exam, labs (eg, lipid panel, lipoprotein (a), hemoglobin A1C), a resting 12-lead electrocardiogram (ECG), exercise treadmill tests with and without ECG, cardiopulmonary exercise testing with and without ECG, resting transthoracic echocardiograms, and/or CAC scores or coronary computed tomography angiography. There are currently no universal PPCS recommendations for all TAs, and screening programs also need to account for the age of individuals and potential occupational hazards (eg the risk of CAD increases with age, so programs may consider a CAC score after a certain age). While TAs have been screened for years with a combination of these various methods, there are very limited data on the diagnostic yield and outcomes from these various screening modalities (Table 1, Number 10). In terms of considering a resting 12-lead ECG among young TA recruits, there are no large-scale studies that have assessed the diagnostic accuracy of contemporary athletic ECG criteria in TAs (most recently published in 2017). It is important to note that while ECG screening among young TAs (eg age<35 years) may be beneficial to detect inherited conditions associated with SCA/D, the utility of resting ECGs for older individuals to test for atherosclerotic CAD remains limited. There is an ongoing pilot program among Air Force, Army, and Navy Service Academies assessing the utility of ECG screening, and the results are eagerly awaited. Cardiorespiratory fitness is an integrated measure of cardiovascular and respiratory health that is predictive of cardiovascular and all-cause mortality. Multiple different TA societies have minimum standards for cardiorespiratory fitness (eg, ≥12 metabolic equivalents of task), most frequently assessed by cardiopulmonary exercise testing or exercise ECG testing, and may restrict an individual from duty until they improve their fitness level. In terms of the diagnostic yield of exercise ECG testing, some societies widely use this screening modality without robust data to support this practice.^8^ Similarly for PPCS with a resting transthoracic echocardiogram, there have been very little data to support this practice. Screening for CAD is enticing among older TAs, given that CAD is a significant risk factor for SCD.^2^^,^^3^ Multiple prior studies have documented that CAC scores may be higher-than-expected in some TAs and a study among military members demonstrated that screening with CAC scores appears cost-effective and provides prognostic value beyond traditional cardiovascular risk factors.^4^^,^^9^ Accordingly, CAC scores are used in screening for CAD in some TA cohorts, such as among astronauts, who are screened with the Astronaut Cardiovascular Health and Risk Modification (Astro-CHARM) risk calculator and CAC scores are repeated every 5 years if initial testing is normal.^10^ There have not been large-scale studies assessing the use of coronary computed tomography angiography in the screening setting or for detailed plaque composition/quantification among TAs. Overall, PPCS has the potential to significantly reduce cardiovascular morbidity/mortality among TAs, but definitive data defining PPCS yield and risk reduction are lacking among TA.

TAs represent unique populations exposed to high physical and psychological stress and potentially severe environmental conditions to ensure the safety of others. Preliminary studies have begun to characterize the incidence of CVD and outcomes among TAs diagnosed with CVD, but our knowledge remains limited. Carefully considered efforts are urgently needed to improve the cardiovascular care of TAs who routinely risk their own lives in service to others.

## Funding support and author disclosures

Dr Petek is compensated as the Medical Director for the Portland Fire Department. Dr Smith is Director of the National Fire Research and Data Center for the U.S. Fire Association, Division of Federal Emergency Management Agency. Dr Kales has served as a paid medical expert witness/examiner in cases involving firefighters and law enforcement. Dr Levine consults with NASA. Drs Franzos and Haigney are employed by the Uniformed Services University of Health Sciences and receive Department of Defense funding for research. All other authors have reported that they have no relationships relevant to the contents of this paper to disclose.
